# Inhibitory Effect of Dihydroaustrasulfone Alcohol on the Migration of Human Non-Small Cell Lung Carcinoma A549 Cells and the Antitumor Effect on a Lewis Lung Carcinoma-Bearing Tumor Model in C57BL/6J Mice

**DOI:** 10.3390/md12010196

**Published:** 2014-01-09

**Authors:** Shuo-Chueh Chen, Yi-Chung Chien, Chun-Hsu Pan, Jyh-Horng Sheu, Chih-Yi Chen, Chieh-Hsi Wu

**Affiliations:** 1 Graduate Institute of Clinical Medical Science, China Medical University, Taichung 404, Taiwan; 2 Division of Pulmonary and Critical Care Medicine, Department of Internal Medicine, China Medical University Hospital, Taichung 404, Taiwan; 3 School of Medicine, China Medical University, Taichung 404, Taiwan; E-Mail: scchen18@gmail.com; 4 School of Pharmacy, China Medical University, 91 Hsueh-Shih Road, Taichung 404, Taiwan; E-Mail: hardway19800710@gmail.com; 5 College of Pharmacy, Taipei Medical University, 250 Wu-Hsing Street, Taipei City 110, Taiwan; E-Mail: joseph.panch@gmail.com; 6 Department of Marine Biotechnology and Resources, National Sun Yat-sen University, 70 Lien-Hai Road, Kaohsiung 804, Taiwan; 7 Division of Chest Surgery and Cancer Center, Department of Surgery, China Medical University Hospital, Taichung 404, Taiwan

**Keywords:** non-small cell lung cancer, migration, tumor, matrix metalloproteinase, marine origin

## Abstract

There are many major causes of cancer death, including metastasis of cancer. Dihydroaustrasulfone alcohol, which is isolated from marine coral, has shown antioxidant activity, but has not been reported to have an anti-cancer effect. We first discovered that dihydroaustrasulfone alcohol provided a concentration-dependent inhibitory effect on the migration and motility of human non-small cell lung carcinoma (NSCLC) A549 cells by trans-well and wound healing assays. The results of a zymography assay and Western blot showed that dihydroaustrasulfone alcohol suppressed the activities and protein expression of matrix metalloproteinase (MMP)-2 and MMP-9. Further investigation revealed that dihydroaustrasulfone alcohol suppressed the phosphorylation of ERK1/2, p38, and JNK1/2. Dihydroaustrasulfone alcohol also suppressed the expression of PI3K and the phosphorylation of Akt. Furthermore, dihydroaustrasulfone alcohol markedly inhibited tumor growth in Lewis lung cancer (LLC)-bearing mice. We concluded that dihydroaustrasulfone alcohol is a new pure compound with anti-migration and anti-tumor growth activity in lung cancer and might be applied to clinical treatment in the future.

## 1. Introduction

Non-small cell lung cancer (NSCLC) is one of the main causes of cancer death, and its incidence is increasing. Surgery, radiotherapy, and chemotherapy are the major treatment methods to reduce lung cancer mortality [1], however, these treatments have harmful side effects on normal healthy cells in the human body. Therefore, it is important to discover new agents to treat lung cancer safely without affecting the body’s healthy cells. The deregulation of signaling pathways such as PI3K/Akt is often implicated in the pathogenesis of NSCLC [2]. Thus, the need for the accelerated development of effective NSCLC therapies is critical. At present, the design of new therapeutic strategies targeting multiple signaling pathways for more effective disease management in NSCLC is a primary focus of current research.

Overgrowth, invasion, and metastasis are the major characteristics of malignancy with poor clinical outcome. Malignant tumor progression depends upon the capacity to invade, metastasize, and promote the angiogenic host response. The dynamics of extracellular matrix (ECM) remodeling have been the focus of intense investigation for many years. The degradative process is mainly mediated by matrix metalloproteinases (MMPs), which are a family of at least 20 zinc-dependent endopeptidases best known for their ability to hydrolyze ECM components [3]. MMP-9 is expressed in large quantities in the human lung cancer cell line A549 and might play an important role in tumor invasion [4]. The activity of MMPs is prone to inhibition by endogenous tissue inhibitor of metalloproteinases (TIMPs), which are specific inhibitors of MMPs, and the imbalance between MMPs and TIMPs may contribute to the degradation or deposition of the ECM [5]. Mitogen-activated protein kinases (MAPKs) play an important regulatory role in cell growth, differentiation, apoptosis, and metastasis [6]. In addition, the phosphatidylinositol-3-kinase/serine/threonine protein kinase (or protein kinase B) (PI3K/Akt) signal transduction pathway is involved in the development, progression, and metastasis of various tumors [7,8,9].

**Figure 1:**
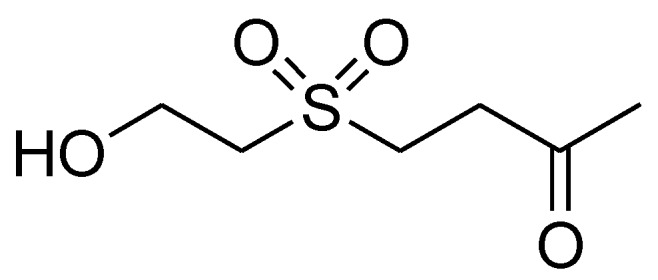
Chemical structure of dihydroaustrasulfone alcohol.

Taiwan is an island and so is surrounded by the sea. In recent studies, the majority of natural marine products have promising biological activities. Jean *et al.* [10,11] showed that natural products isolated from Taiwanese soft corals, such as lemnalol and capnellene, are useful for the treatment of inflammatory diseases in rats. In some studies, the investigation of bioactive marine natural products has led to the isolation of compounds with neuroprotective [12] and anti-inflammatory [13] activities from soft corals. Gallet *et al.* [14] indicated that cancer medication and radiotherapy can trigger an inflammatory response. This inflammation is characterized by an increase of cytokines, angiogenic factors, adhesion molecules, and matrix metalloproteinases (MMPs) [15,16]. It has also been shown that chronic inflammation could increase the risk of developing several types of cancer [17]. In previous studies, dihydroaustrasulfone alcohol (Figure 1) produced *in vitro* anti-inflammatory activity. Wen *et al.* [18] showed that dihydroaustrasulfone alcohol not only exhibited *in vitro* anti-inflammatory activity but also showed potent therapeutic ability in the treatment of neuropathic pain, atherosclerosis, and multiple sclerosis in rats. The anti-metastatic effect of dihydroaustrasulfone alcohol in human NSCLC A549 cells is still unclear. In the present study, we investigated the anti-metastatic effects and underlying mechanisms of dihydroaustrasulfone alcohol in the A549 cell line.

## 2. Results and Discussion

### 2.1. Cytotoxicity of Dihydroaustrasulfone Alcohol in A549 Cells

**Figure 2:**
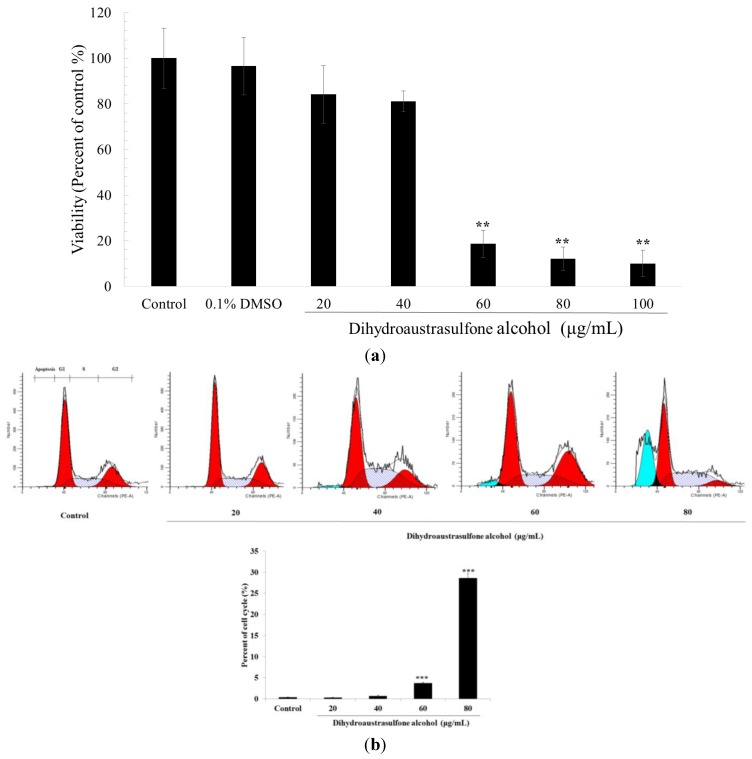
.Cytotoxicity of dihydroaustrasulfone alcohol to A549 cells. (**a**) Viability of A549 cells incubated with dihydroaustrasulfone alcohol (20, 40, 60, 80 or 100 μg/mL) for 24 h. Cell viability was measured using an MTT assay and is expressed as the % of cell survival relative to the control, which means sample without drug treatment as all of the results. (**b**) Flow cytometric analysis of the effect of dihydroaustrasulfone alcohol on the cell cycle of A549 cells. The cells were treated with dihydroaustrasulfone alcohol at concentrations of 20, 40, 60 or 80 μg/mL for 24 h. The value on the x-axis represents the DNA content, while the shaded area indicates the percentage of cells at the S phase, blue area indicate sub-G1 phase, and red areas indicate G1 phase (left) and G2 phase (right), individually. This graph shows the percentage of sub-G1 contents in A549 cells treated with dihydroaustrasulfone alcohol. The values are the means of three separate experiments, with the standard deviation represented by vertical bars. * *P* < 0.05; ** *P* < 0.01; *** *P* < 0.001.

3-(4,5-Dimethylthiazol-2-yl)-2,5-diphenyltetrazolium bromide (MTT) assay is broadly used to test cell cytotoxicity. Tsai *el al*. [19] found that a novel anti-cancer drug, MPT0B014, exhibited potent anti-proliferative activity against human NSCLC cell lines using MTT assay. Wyrebska *et al.* [20] also used MTT assay to demonstrate cytotoxicity of a new synthetic α-methylene-δ-lactones against breast cancer cell lines. To determine whether dihydroaustrasulfone alcohol decreases cancer cell viability, A549 cells were screened using the MTT assay for cell cytotoxicity in the presence of different concentrations of dihydroaustrasulfone alcohol for 24 h. As shown in Figure 2a, dihydroaustrasulfone alcohol significantly inhibited the viability of A549 cells in a concentration-dependent manner (IC_50_ = 0.273 mM). Because the MTT assay showed that dihydroaustrasulfone alcohol at 60, 80, and 100 μg/mL significantly suppressed cell viability, we postulated that the inhibitory effects of dihydroaustrasulfone alcohol on cell viability might be mediated by apoptosis. Therefore, the effect of dihydroaustrasulfone alcohol concentration on the cell cycle and apoptosis was evaluated at 20, 40, 60, or 80 μg/mL (Figure 2b). The results demonstrated that treatment for 24 h with dihydroaustrasulfone alcohol at 20 and 40 μg/mL had no effect on apoptosis in the sub-G1 phase (Figure 2b). Therefore, the concentrations 20, 30, and 40 μg/mL were selected for subsequent studies.

### 2.2. Effect of Dihydroaustrasulfone Alcohol on a Wound-Healing Assay in A549 Cells

**Figure 3:**
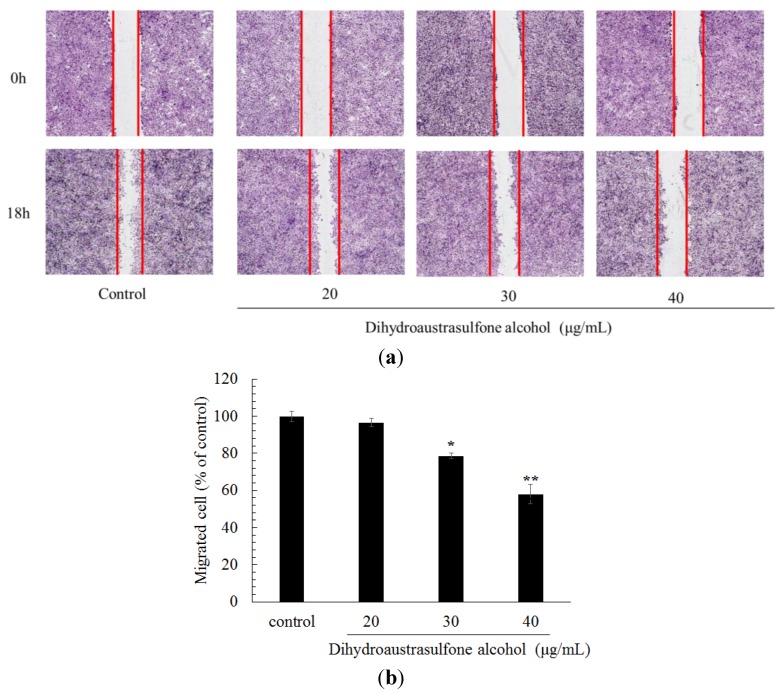
Effects of dihydroaustrasulfone alcohol on the wound healing migration of A549 cells. (**a**) A wound was introduced by scraping confluent cell layers with a pipet tip. A549 cells were incubated with dihydroaustrasulfone alcohol (20, 30, or 40 μg/mL) for 18 h, and the migration distances of the cells were calculated. Representative photographs of invading cells that received either control or dihydroaustrasulfone alcohol treatment. (**b**) Cell that migrated across the red lines were counted in six random fields in each treatment. The mean number of cells in the denuded zone was quantified in three independent experiments. The values (means ± SD, *n* = 3) differed significantly (* *P* < 0.05; ** *P* < 0.01).

Wound healing assay were broadly used in research focused on cancer cell migratory ability inhibition. Chung *et al.* [21] reported that marine algal fucoxanthin markedly suppressed highly metastatic murine B16-F10 melanoma cell migration and invasion in wound healing and trans-well assay. To evaluate the effect of dihydroaustrasulfone alcohol on the migration of lung cancer cells, we used a trans-well assay and a wound healing assay. For the latter, the confluent monolayer was scraped with a sterile micropipette tip to create a scratch wound. We found that dihydroaustrasulfone alcohol added at 30 and 40 μg/mL significantly decreased the migration of A549 cells (Figure 3).

### 2.3. Effect of Dihydroaustrasulfone Alcohol in a Trans-well Assay with A549 Cells

**Figure 4:**
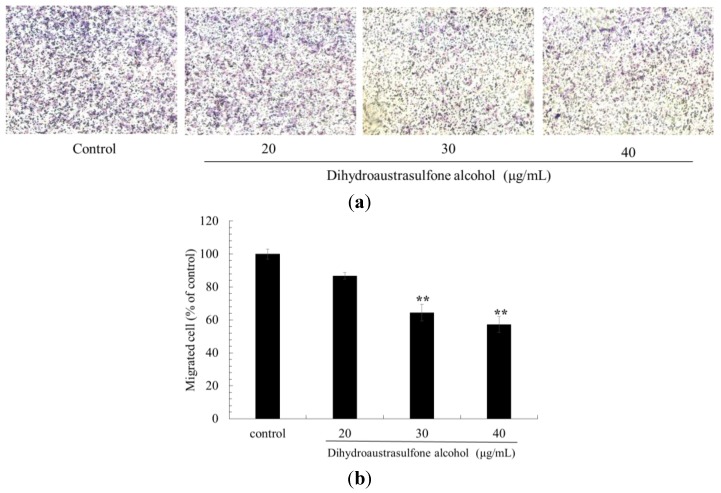
Effects of dihydroaustrasulfone alcohol on the trans-well migration assay of A549 cells. (**a**) A549 cells were incubated with dihydroaustrasulfone alcohol (20, 30 or 40 μg/mL) for 18 h, and the (**b**) trans-well migration of cells was calculated. Photos of the migration of A549 cells were taken under a microscope (200-fold magnification). The values (means ± SD, *n* = 3) differed significantly (* *P* < 0.05; ** *P* < 0.01).

Cancer cell migration inhibition was also commonly studied through trans-well assay. Luo *et al.* [22] declared that overexpression of MicroRNA-497 inhibited breast cancer cellular migration and invasion, which were documented by trans-well assay. Liu *et al.* [23] also showed that inhibition of long non-coding RNA HOTAIR by RNA interference (RNAi) decreased the migration and invasion of NSCLC cells, which was also proved by trans-well assay. The trans-well assay was also used to investigate the migration of A549 cells after dihydroaustrasulfone alcohol treatment. The results showed that treatment with 30 or 40 μg/mL dihydroaustrasulfone alcohol significantly decreased migration (Figure 4).

### 2.4. Effect of Dihydroaustrasulfone Aalcohol Inhibits the Release of MMP in A549 Cells

**Figure 5:**
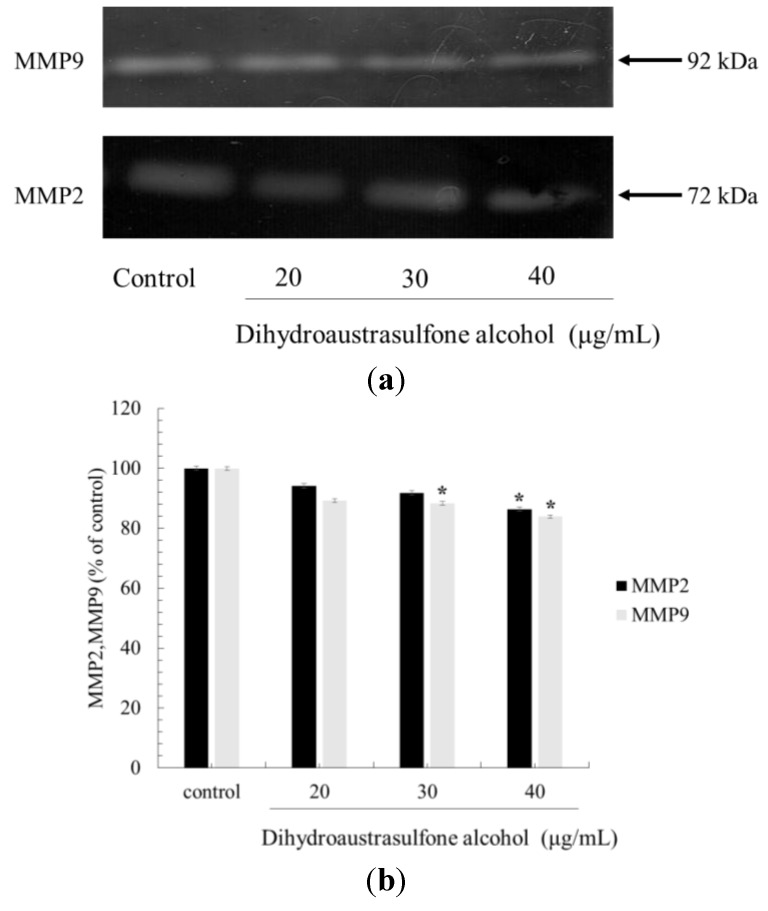
Effects of dihydroaustrasulfone alcohol on the MMP-2 and MMP-9 activities of A549 cells. (**a**) The cells were treated with various concentrations (20, 30, or 40 μg/mL) of dihydroaustrasulfone alcohol for 24 h. The conditioned media were collected, and MMP-2 and MMP-9 activities were determined by gelatin zymography. (**b**) The activities of these proteins were subsequently quantified by densitometric analysis. The values (means ± SD, *n* = 3) differed significantly (* *P* < 0.05).

MMP-2 and MMP-9 were well studied as cancer cell migration and invasion related proteins. Zymography assay is a common method to detect their activities. Wyrebska A *et al.* [20] reported a new synthetic α-methylene-δ-lactones with cytotoxicity, inhbibition migration and invasion activity against two breast cancer cell lines. They indicated that the decreased secretion of enzymes responsible for the degradation of the extracellular matrix, MMP-9 and uPA through zymography assay. Zhu *et al.* [24] also demonstrated that alternol inhibits migration activity of hepatocellular carcinoma cell line by MMP-9 suppression using zymography assay. In our study, through a zymography assay, we also found that dihydroaustrasulfone alcohol significantly inhibited the activities of MMP-2 and MMP-9 (Figure 5). These results demonstrated that the anti-metastatic effect of dihydroaustrasulfone alcohol was associated with the inhibition of enzymatically degradative processes of cancer cell migration. To our knowledge, this is the first study to demonstrate that dihydroaustrasulfone alcohol reduces the biochemical mechanisms of migration in A549 cells.

### 2.5. Dihydroaustrasulfone Alcohol Inhibits Migration-Related Proteins and PI3K/AKT Signaling in A549 Cells

**Figure 6:**
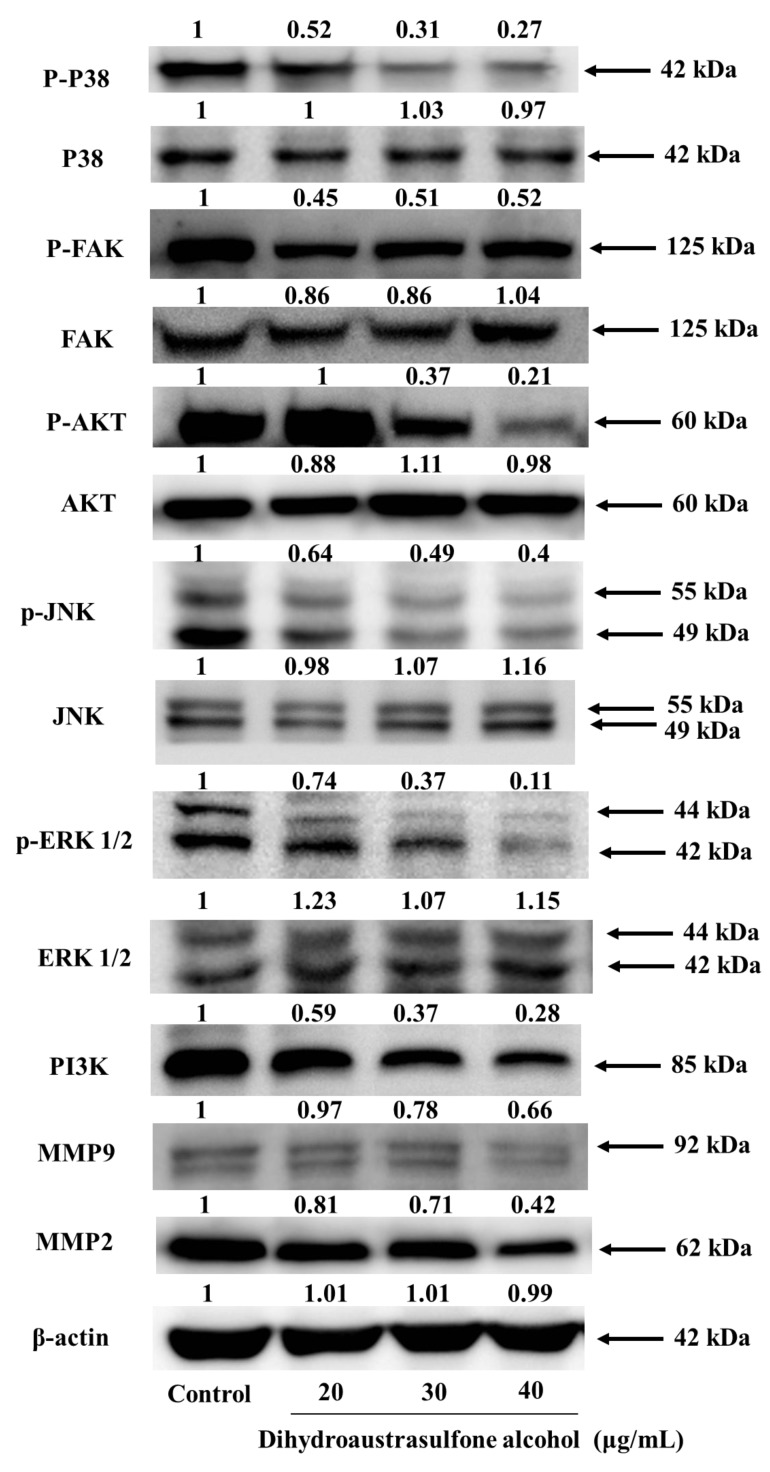
Effects of dihydroaustrasulfone alcohol on migration-related proteins and PI3K/AKT signaling in A549 cells. A549 cells were treated with 20, 30, or 40 μg/mL, and cell lysates were subjected to SDS-PAGE and Western blotting before quantification by densitometric analyses (using the control as 100%). The values indicate the density proportion of each protein compared with the control.

**Figure 7:**
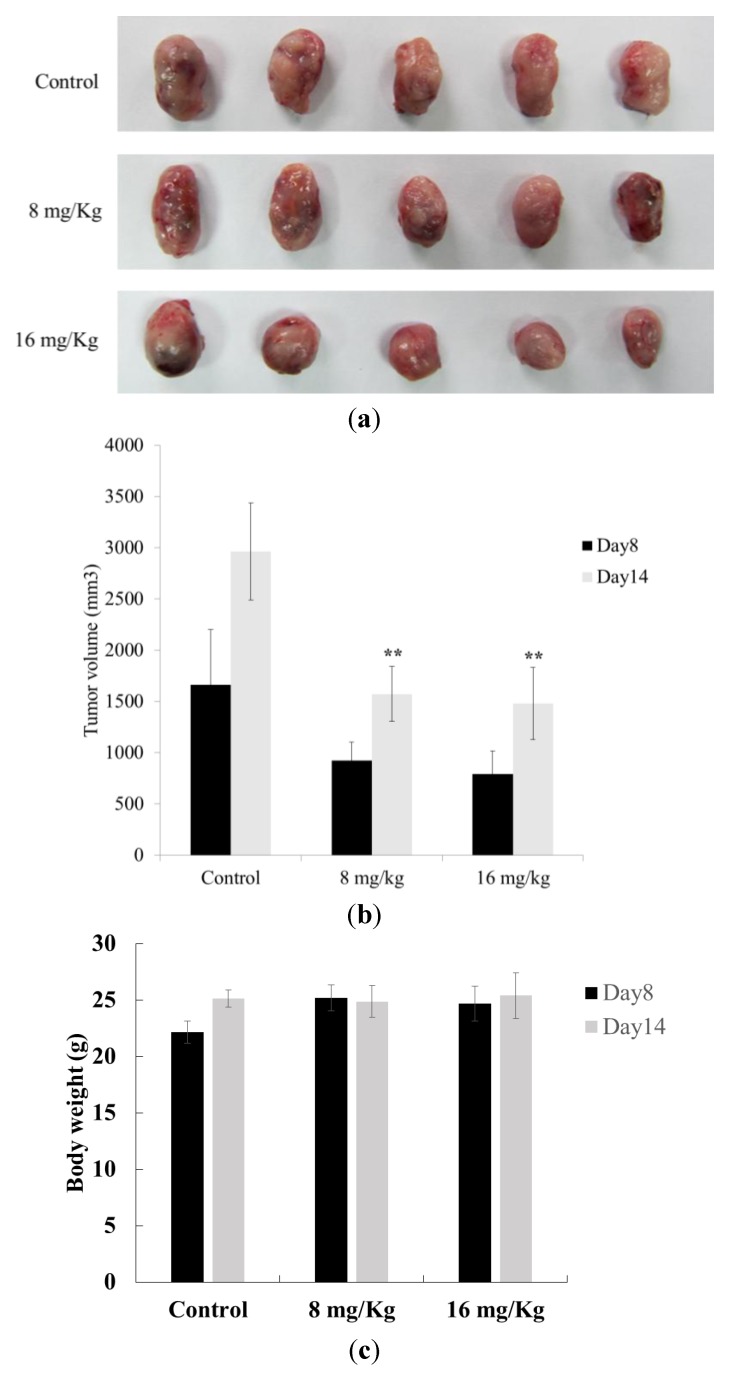
Antitumor effect of dihydroaustrasulfone alcohol on a Lewis lung carcinoma-bearing tumor model in C57BL/6J mice. Eighteen C57BL/6J mice were randomly divided into 3 groups. (**a**) Representative subcutaneous tumor mass of mice, (**b**) solid tumor volume, and (**c**) body weight from each group are shown. The data are presented as the mean ± S.D. of six animals at days 8 and 14 after tumor implantation. ** indicates *p* < 0.01 compared with control group (treated with normal saline) of each group.

Certain tumor cells become motile and attack the host tissue, leading to metastasis during cancer progression. Focal adhesion kinase (FAK) plays an important role in cancer cell proliferation and metastasis because it can be activated in response to various stimuli [25]. According to recent research, FAK regulates focal adhesion signaling by phosphorylating various substrates and by acting as a scaffold for protein-protein interactions and MMP-mediated matrix degradation, which consecutively regulates downstream signaling cascades [26]. FAK, a cytoplasmic kinase that is involved in the ECM and integrin-mediated signaling pathways, has been hypothesized to have an essential role in metastasis through the modulation of tumor cell migration and invasion [27]. Integrin-mediated FAK signaling occurs through the phosphorylation of Tyr397. Particularly, phosphorylation of Tyr397 forms a high-affinity binding site for the Src-homology domain 2 (SH2) domains of Src family kinases, therefore promoting Src kinase activity. Activated FAK in cancer cells relays signals through multiple downstream targets. For example, activated FAK binds the SH2 of PI3K, thereby transporting the catalytic subunit of PI3K to the membrane, where it catalyzes the phosphorylation of inositol lipids in lung cancer cell migration [28]. The residues surrounding Tyr397 can also constitute a sequence that binds to the Ras signaling pathway. The downstream targets of the Ras signaling pathway include ERK1/2 [29]. Indeed, these pathways are activated during integrin binding to the ECM, resulting in the transduction of external stimuli from the ECM to the nucleus [30]. Certain results suggest that there is an anti-invasive effect in androgen-independent prostate cancer that occurs through the control of MMP-9 expression through the suppression of the EGFR/JNK pathway [31]. FAK and MAPK signaling have been shown to be involved in MMP-2 secretion by QG90 lung cancer cells [32]. Moreover, the MMP-9 gene promoter is partially regulated through activation of the ERK1/2 pathway [33]. A recent study showed that p38 is implicated in mediating bladder cancer invasion via the regulation of MMP-2 and MMP-9 through mRNA stability [34]. Additionally, p38γ MAPK activates c-Jun, and activated c-Jun recruits p38γ to the MMP-9 promoter as a cofactor to induce MMP-9 promoter trans-activation and cell invasion [35]. In this study, we found that dihydroaustrasulfone alcohol inhibited the activation of FAK, as evidenced by reduced phosphorylation of FAK (Figure 6). We also demonstrated that treatment with dihydroaustrasulfone alcohol inhibited the phosphorylation of ERK1/2 and JNK1/2 (Figure 6). In addition, we showed that dihydroaustrasulfone alcohol inhibited PI3K/AKT in A549 cells. Thus, it appears that FAK promotes A549 cancer cell migration in concert with the activation of the PI3K/AKT signaling pathways. Increased phosphorylation of FAK and its downstream targets ERK1/2, PI3K, and AKT have been shown in A549 cells stimulated by fibronectin. Several studies have indicated that FAK/PI3K/Akt is involved in the regulation of MMP-2 and MMP-9 activities in different cell types [7,36]. To assess whether dihydroaustrasulfone alcohol inhibits the phosphorylation of FAK, AKT, and the protein level of PI3K, A549 cells were treated with various concentrations of dihydroaustrasulfone alcohol (20, 30, or 40 μg/mL). Figure 6 shows dihydroaustrasulfone alcohol inhibited the activation of FAK and AKT through a decrease in the phosphorylation of FAK and AKT. In addition, dihydroaustrasulfone alcohol inhibited the protein levels of PI3K in a dose-dependent manner (Figure 6). Zeng *et al.* [37] reported that activated FAK-induced PI3K is required for the production of matrix metalloproteinases (MMPs). PI3K is one of the critical downstream signal molecules of FAK pathways [38]. Therefore, our results demonstrate that dihydroaustrasulfone alcohol inhibited the expressions of p-FAK, pAKT, and PI3K. In addition, Shih *et al.* [9] reported that the PI3K/AKT signal transduction pathway regulates cell invasion and metastasis of NSCLC and is closely associated with the development and progression of various tumors. Based on our results, the proposed signaling pathways are shown in Figure 7 for the dihydroaustrasulfone alcohol-induced inhibition of A549 cell migration.

### 2.6. Antitumor Effect of Dihydroaustrasulfone Alcohol on a Lewis Lung Carcinoma (LLC)-Bearing Tumor Model in C57BL/6J Mice

*In vivo* study is necessary to proof anti-tumor growth effect of the test drug. Zhou *et al.* [39] presented that combination of Epigallocatechin gallate (EGCG) and curcumin exhibited tumor growth suppression by the combination of these two agents in a xenograft nude mice model using A549 human lung adenocarcinoma cell line. Kim *et al.* [40] also reported galbanic acidintra-peritoneal injection treatment inhibited LLC-induced angiogenesis and inhibited the LLC allograft in syngeneic mice tumor growth. In our *in vivo* study, we investigated the antitumor effect of dihydroaustrasulfone alcohol in a Lewis lung carcinoma-bearing tumor model in C57BL/6J mice. The results showed that a 14-day treatment of 8 or 16 mg/kg dihydroaustrasulfone alcohol significantly reduced tumor size compared with the LLC control group (Figure 7). These data also show that the body weights of the mice that received treatment were not significantly different from the LLC control group at days 8 or 14 (Figure 7c).

## 3. Experimental Section

### 3.1. Materials

**Scheme 1:**
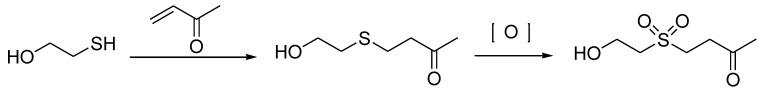
Synthetic sequence of dihydroaustrasulfone alcohol.

Dihydroaustrasulfone alcohol is the synthetic precursor of austrasulfone, which is a marine natural product possessing neuroprotective activity. Dihydroaustrasulfone alcohol was prepared by the reaction of 2-mercaptoethanol with methyl vinyl ketone to produce the corresponding sulfide, followed by oxidation of this sulfide with *m*-chloroperoxybenzoic acid (Scheme 1) [18]. 3-(4,5-Dimetylthiazol-2-yl)-2,5-diphenyltetrazolium bromide (MTT), RNase A, propidium iodide (PI), trypsin, BSA, Tween-20, -80 and DMSO were purchased from Amresco Inc. (Solon, OH, USA). F-12 and fetal bovine serum (FBS) were purchased from GIBCO BRL (Rockville, MD, USA). Cell culture supplies were purchased from Costar (Corning, Inc., Cypress, CA, USA). The antibodies against AKT, Rac-1, MAPK/extracellular signal-regulated kinase (ERK) 1/2, c-Jun NH_2_-terminal kinase (JNK)/stress-activated protein kinase, and p38 MAPK proteins and phosphorylated proteins were purchased from Cell Signaling Technology (Beverly, MA, USA). Anti-ERK1/2, anti-PI3K, antifocal adhesion kinase (FAK), anti-p-FAK, and horseradish peroxidase-conjugated goat antimouse IgG antibodies were purchased from Santa Cruz Biotechnology Co. (Santa Cruz, CA, USA). β-actin was purchased from Chemicon (Temecula, CA, USA).

### 3.2. Cell Lines and Cultures

Human NSCLC A549 cells were obtained from Food Industry Research and Development Institute (Hsinchu, Taiwan). A549 cells were cultured in F-12 medium (Gibco BRL, Rockville, MD, USA) containing 10% fetal bovine serum (FBS) (Gibco BRL, Rockville, MD, USA), 100 U/mL penicillin, and 100 mg/mL streptomycin (Gibco BRL, Rockville, MD, USA) at 37 °C in a humidified atmosphere comprised of 95% air and 5% CO_2_. In all of the experiments, the medium was supplemented with 1% (v/v) fetal bovine serum (FBS). LLC cells were obtained from the American Type Culture Collection (ATCC; CRL-1642) and maintained as monolayer cultures in DMEM supplemented with 10% (v/v) FBS, 1% non-essential amino acids and 1% sodium pyruvate. All cells were incubated in a humidifier with 5% CO_2_ at 37 °C.

### 3.3. Cell Proliferation Assay

The MTT assay was performed in the A549 cell lines to measure the cytotoxicity of dihydroaustrasulfone alcohol. All cell lines were seeded in 96-well plates with 2 × 10^4^ cells/well in culture medium. Dihydroaustrasulfone alcohol was dissolved in PBS. The cells were treated with various concentrations of dihydroaustrasulfone alcohol as indicated in each figure. After 24 h, the number of viable cells was determined. Then, 5 mg/mL MTT was added to each well, and the plate was incubated at 37 °C for 3 h. The medium was removed, and 50 μL DMSO was added. The absorbance at 590 nm was measured for each well on an ELISA reader. The data are presented as the mean ± SEM of three independent experiments.

### 3.4. Flow Cytometry Analysis

A549 cells (2 × 10^5^) were seeded into each well of a 12-well plate (TPP; Techno Plastic Products AG, Trasadingen, Switzerland) 24 h before being treated with various concentrations of dihydroaustrasulfone alcohol for 24 h. The cells were harvested with trypsin-EDTA, washed twice with 10 mL ice-cold PBS, fixed in 70% (v/v) ethanol, and kept at 4 °C. The cells were then stained with propidium iodide (PI) [100 μg/mL PI, 0.2% (v/v) Nonidet P-40, and 1 mg/mL RNase A (DNase-free) in PBS lacking Ca2+ and Mg2+ at a 1:1:1 ratio by volume] and the DNA contents were analyzed with flow cytometry (Becton Dickinson, San Jose, CA, USA). The intensity of PI fluorescence was linearly amplified, and both the area and width of the fluorescence pulse were measured. Ten thousand events were acquired, and the percentage of hypodiploid (apoptosis, sub-G1) events and the percentages of cells in the G0/G1, S and G2/M phases were determined using the DNA analysis software ModFitL T, version 2.0 (Verity Software, Topsham, ME, USA).

### 3.5. Wound Healing Assay

For cell motility determination, A549 cells (2 × 10^5^ cells/mL) were seeded in a 12-well tissue culture plate. After one day, the center of the cell monolayers was scraped with a sterile micropipette tip to create a straight zone (gap) of constant width. Then, each well was washed with PBS, and A549 cells were exposed to various concentrations of dihydroaustrasulfone alcohol (20, 30 or 40 μg/mL). Wound closure was monitored and photographed at 0 h and 18 h with a Nikon inverted microscope. To quantify the migrated cells, pictures of the initial wounded monolayers were compared with the corresponding pictures of cells at the end of the incubation. Artificial lines fitting the cutting edges were drawn on pictures of the original wounds and overlaid on the pictures of cultures after incubation. Cells that had migrated across the white lines were counted in six random fields from each triplicate treatment.

### 3.6. Cell Migration Assay

Tumor cell migration was assayed in trans-well chambers (Millipore) according to the method reported by Huang *et al.* [29], with some modifications. Briefly, trans-well chambers with 6.5 mm polycarbonate filters of 8 μm pore size were used. A549 cells (1 × 10^4^ cells/mL) and 20, 30 or 40 μg/mL of dihydroaustrasulfone alcohol were suspended in F-12 (100 μL, serum free), placed in the upper trans-well chamber, and incubated for 24 h at 37 °C. Then, the cells on the upper surface of the filter were completely wiped away with a cotton swab, and the lower surface of the filter was fixed in methanol, stained with 10% Giemsa solution, and counted under a microscope at a magnification of 200×. For each replicate, the tumor cells in 10 randomly selected fields were determined and the counts were averaged.

### 3.7. Determination of MMP-2 and MMP-9 by Zymography

MMPs in the medium released from A549 cells were assayed using gelatin zymography (8% zymogram gelatin gels) according to the methods reported by Huang *et al.*, with some modification [29,41]. Briefly, the culture medium was electrophoresed (80 V for 120 min) in an 8% SDS-PAGE gel containing 0.1% gelatin. The gel was then washed at room temperature in a solution containing 2.5% (v/v) Triton X-100 with two changes and subsequently transferred to a reaction buffer for enzymatic reaction containing 1% NaN_3_; 10 mM CaCl_2_; and 40 mM Tris-HCl, pH 8.0; at 37 °C with shaking overnight (for 12–15 h). Finally, the MMP gel was stained for 30 min with 0.25% (w/v) Coomassie blue in 10% acetic acid (v/v) and 20% methanol (v/v) and destained in 10% acetic acid (v/v) and 20% methanol (v/v).

### 3.8. Western Blotting Analysis

A549 cells were plated in 10-cm dishes at a density of 3 × 10^6^ cells per dish and incubated with 20, 30 or 40 μg/mL dihydroaustrasulfone alcohol in F-12 containing 1% (v/v) FBS for 24 h. The cells were collected, lysed in a lysis solution, and then incubated at 25 °C for 10 min. Total proteins were separated with SDS-PAGE before being transferred to PVDF membranes. The membranes were blocked with 5% (v/v) nonfat dry milk in PBS-Tween 20 for one hour and changed to an appropriate dilution of specific primary antibodies in TBS-T buffer overnight at 4 °C. The blots were then incubated with horseradish peroxidase-linked secondary antibody for 1 h and then developed with the electrochemiluminescence (ECL) reagent and exposed to Hyperfilm (Amersham, Arlington Height, IL, USA). The data were analyzed by Gel-Logic 200 Imaging Systems, Molecular Imaging Software.

### 3.9. Lewis Lung Cancer Cell Bearing Tumor Model of C57BL/6J Mice

The animal experiments were approved by the Institutional Animal Care and Use Committee of China Medical University (approval ID: 102-194-C). All animal care followed the institutional animal ethical guidelines of China Medical University. C57BL/6J male mice (average 25–28 g; 6 weeks old) were obtained from the Laboratory Animal Center, College of Medicine, National Taiwan University (Taipei, Taiwan). The C57BL/6J mice were kept on a 12 h light/dark cycle at 25 °C. C57BL/6J mice were subcutaneously implanted with LLC cells (7 × 10^5^ cells in 100 μL). The treatment of dihydroaustrasulfone alcohol start while all the tumors volume were larger than 100 mm^3^. Eighteen mice were randomly assigned to three treatment groups (6 animals per group). Group I (untreated group) was subcutaneously grafted with LLC cells and given vehicle solvent. Group II was grafted with LLC cells and then treated with dihydroaustrasulfone alcohol (8 mg/kg). Group III was grafted with LLC cells and then treated with dihydroaustrasulfone alcohol (16 mg/kg). The dihydroaustrasulfone alcohol was diluted in normal saline and administered by IP. Body weight and tumor volume were measured every week after cell injection. The tumor width (W) and length (L) were measured every week by calipers, and the tumor volume was calculated as L × W2 × 0.52 [42]. The mice were treated daily for 2 weeks and then sacrificed.

### 3.10. Statistical Analysis

The values are presented as the mean ± SD relative to the control values. Statistically significant differences from the control group were identified by one-way ANOVA for the data. *P* < 0.05 was considered statistically significant for all tests.

## 4. Conclusions

**Figure 8:**
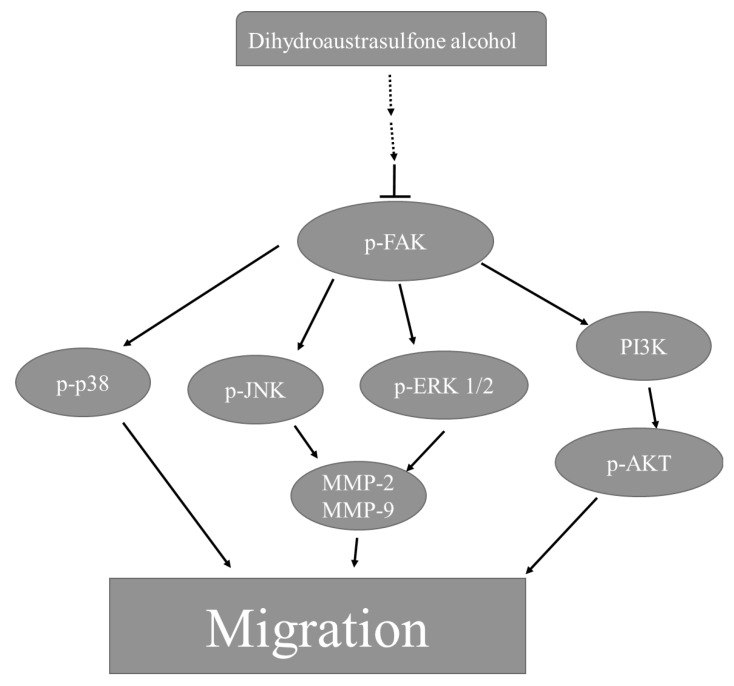
Proposed signaling pathways for dihydroaustrasulfone alcohol-mediated inhibition of A549 cell migration. The effect of dihydroaustrasulfone alcohol is most likely achieved through the inhibition of FAK, which regulates MMP-2 expression through the MAPK and PI3K/AKT signaling pathways.

In conclusion, we have demonstrated that dihydroaustrasulfone alcohol inhibits the activities of MMP-2 and MMP-9 and the migration of human NSCLC cell line (Figure 3, Figure 4 and Figure 5). Mechanistically, we showed that migration inhibition induced by dihydroaustrasulfone alcohol may occur through inactivation of the ERK1/2 signaling pathway, exerting inhibitory effects on phospho-FAK protein expression and inhibiting PI3K and phospho-Akt levels, thereby decreasing the activities of MMP-2 and MMP-9 in A549 cells (Figure 6 and Figure 8). Dihydroaustrasulfone alcohol also markedly inhibited tumor growth in Lewis lung cancer (LLC)-bearing mice (Figure 7). Therefore, our data demonstrate that dihydroaustrasulfone alcohol has a potential of anti-cancer effect and should perform more research focused on other mechanism other than migration inhibition.
